# “A Phenomenal Person and Doctor”: Thank You Letters to Medical Care Providers

**DOI:** 10.2196/ijmr.7107

**Published:** 2017-11-02

**Authors:** Talya Miron-Shatz, Stefan Becker, Franklin Zaromb, Alexander Mertens, Avi Tsafrir

**Affiliations:** ^1^ Center for Medical Decision Making Faculty of Business Administration Ono Academic College Kiryat Ono Israel; ^2^ Center for Medicine in the Public Interest New York, NY United States; ^3^ Institute for Drug Safety University Hospital Essen Essen Germany; ^4^ Department of Nephrology University Duisburg-Essen Essen Germany; ^5^ National Authority of Measurement and Evaluation in Education Ramat Gan Israel; ^6^ Institute of Industrial Engineering and Ergonomics Aachen University Aachen Germany; ^7^ Department of Obstetrics and Gynecology Shaare Zedek Medical Center Hebrew University of Jerusalem Jerusalem Israel

**Keywords:** quality of health care, doctor-patient relationship, content analysis, decision making, doctor-patient communication, clinical encounter, patient satisfaction, online reviews, patient-centered care

## Abstract

**Background:**

Thank you letters to physicians and medical facilities are an untapped resource, providing an invaluable glimpse into what patients notice and appreciate in their care.

**Objective:**

The aim of this study was to analyze such thank you letters as posted on the Web by medical institutions to find what patients and families consider to be good care. In an age of patient-centered care, it is pivotal to see what metrics patients and families apply when assessing their care and whether they grasp specific versus general qualities in their care.

**Methods:**

Our exploratory inquiry covered 100 thank you letters posted on the Web by 26 medical facilities in the United States and the United Kingdom. We systematically coded and descriptively presented the aspects of care that patients and their families thanked doctors and medical facilities for. We relied on previous work outlining patient priorities and satisfaction (Anderson et al, 2007), to which we added a distinction between global and specific evaluations for each of the already existing categories with two additional categories: general praise and other, and several subcategories, such as treatment outcome, to the category of medical care.

**Results:**

In 73% of the letters (73/100), physicians were primarily thanked for their medical treatment. In 71% (71/100) of the letters, they were thanked for their personality and demeanor. In 52% cases (52/100), these two aspects were mentioned together, suggesting that from the perspective of patient as well as the family member, both are deemed necessary in positive evaluation of medical care. Only 8% (8/100) of the letters lacked reference to medical care, personality or demeanor, or communication. No statistically significant differences were observed in the number of letters that expressed gratitude for the personality or demeanor of medical care providers versus the quality of medical care (χ21, N=200=0.1, not statistically significant). Letters tended to express more specific praise for personality or demeanor, such as being supportive, understanding, humane and caring (48/71, 68%) but more general praise for medical care (χ21, N=424=63.9, P<.01). The most often mentioned specific quality of medical care were treatment outcomes (30/73, 41%), followed by technical competence (15/73, 21%) and treatment approach (14/73, 19%). A limitation of this inquiry is that we analyzed the letters that medical centers chose to post on the Web. These are not necessarily a representative sample of all thank you letters as are sent to health care institutions but are still indicative of what centers choose to showcase on the Web.

**Conclusions:**

Physician demeanor and quality of interaction with patients are pivotal in how laymen perceive good care, no less so than medical care per se. This inquiry can inform care providers and medical curricula, leading to an improvement in the perceived quality of care.

## Introduction

Patient satisfaction is imperative to the practice of medicine. Indeed, some elements of the therapeutic process, such as patients’ adherence to medication, are related not only to the medical aspects of care but also to whether the patient feels acknowledged and appreciates that the doctor sees him as a person [1]. Patient-centeredness is now deemed as central to good medical care, such that we now view the process as a therapeutic dyad [2]. This study aims to answer the question of what patients value in the medical care they receive by the analysis of thank you letters to physicians and medical centers, as posted on the Web.

The act of writing these letters should not be taken for granted, as “less than 3% of those who have visited a hospital Facebook page or even liked the hospital Facebook page care to comment on a hospital post or share thoughts or express emotions...” [3], and patients’ thank you notes constituted only 7% of the comments on US hospitals’ Facebook pages. We interpret this as signifying that thank you notes are fairly rare and nontrivial, thus highlighting the reason to explore them in depth.

### Previous Work

Anderson and colleagues performed content analysis of 2917 patients’ comments on their health care experiences with a rated doctor or a clinic, as submitted to the patient satisfaction survey at DrScore website [4]. In their taxonomy of the main elements of patients’ experiences with health care providers, Anderson and colleagues identified 25 categories of health care quality valued by patients that mapped onto the following seven broad thematic categories:

- Access to physician or health care services
- Communication with and by provider
- Personality and demeanor of provider, such as the extent to which the physician was supportive, caring, and compassionate
- Quality of medical care processes such as diagnosis and treatment
- Continuity of care as related to follow-up on health care issues or concerns, making referrals, and discussing test results
- Quality of health care facilities
- Office staff

### Choice of Anderson Taxonomy

This taxonomy offers categories that are suitable for capturing and distinguishing among a wide range of both positive and negative patient feedback relating to their health care experiences. By comparison, there are other existing surveys that are commonly used in both the United States and the United Kingdom, such as the Medical Interview Satisfaction Scale (MISS) [5,6] that examines patients’ satisfaction with their medical interview and the Consultation Satisfaction Questionnaire (CSQ) [7]. These examine the clinical encounter and assess patient satisfaction with individual doctor-patient consultations. Both those surveys assess more narrowly defined scopes of patient perceptions of health care [8]. Therefore, we chose to use the Anderson taxonomy [4] with various modifications, as listed in the Methods section.

### Research Questions

Patient evaluations and the measures capturing them can be narrow or broad. This determines how indicative they are of merit or flaw. As noted by Cleary and McNeil, “global measures of satisfaction are affected by many factors, and unless a study is conducted in extremely well-controlled circumstances, it is difficult to interpret global measures” [9]. This implies that measurement of specific care domains is to be preferred over the measurement of broader ones.

The shared decision-making paradigm has been an emerging priority in medical parlance and practice [10,11]. The category of communication as included in our study allows for examination of aspects of the doctor-patient interaction, and the degree to which the patient receives information, is listened to, and—as a culmination of shared decision making—is considered a partner.

In the spirit of a call for exercising neutrality when examining and assessing medical quality [12], we posed two questions. Our main research question for this exploratory, small-scale investigation was as follows: what categories and aspects do patients mention in their thank you letters? The premise—which we cannot experimentally validate—is that patients mention the things they appreciate. Another research question was whether the patients were referring to general or specific aspects within categories [9,12,13].

## Methods

### Selection of Letters

The sample consisted of thank you letters and brief testimonials from patients or their friends and family (for the sake of brevity, we will continue with “patients”) to various medical centers located throughout the United States and the United Kingdom and posted on the Web by the centers. These letters and testimonials were obtained using Google’s search engine by entering the following combination of terms: “medical center patient thank-you letters” and “medical center patient testimonials.” These search terms retrieved 89,800,000 results and 7,950,000 results, respectively. This search was performed in early December 2015.

As this was designed as an exploratory investigation, we planned to only analyze 100 letters. Therefore, we limited our content sample to postings from 26 US or UK medical facilities that, taken together, provided a wide range of medical services to their patients and that appeared in the first 10 pages of search results. We did not include sources that only posted brief letter excerpts or quotes from patients. As the main research question was—what aspects of care do patients thank doctors and medical facilities for—we sought to collect complete letters and testimonials.

On the basis of the order of appearance in search results, we chose medical facilities subject to the constraint that they represented the different types of medical institutions (university medical centers, community or regional hospitals, or specialty clinics) and locations (the United States or the United Kingdom) in equal proportion to the total number of Internet postings retrieved in our Internet search corresponding to the various types of medical institutions and locations. We then retrieved 100 letters in order of appearance from the medical facilities’ Internet sites for coding and content analysis.

The study only analyzed publicly available materials that had already been posted on the Web by medical facilities. This is akin to analyzing newspaper articles. We neither contacted the letter writers nor did we have identifying information about them. Therefore, we did not require approval from the institutional review board.

Table 1 summarizes the characteristics of thank you letters in the content analysis. The final sample of 100 letters included 572 sentences and 773 utterances (ie, a bit of spoken, or in the case of letters, written language, which conveys a message). For example, the following sentence consists of two utterances: I arrived at the clinic in the morning (1) and found it very clean. (2). The letters and testimonials included in the study were posted by the medical centers themselves, suggesting the possibility of a selection bias in what they chose to present. That said, this study illuminates the content of letters that were posted on the Web but not all types of thank you letters ever sent to physicians and medical centers.

**Table 1:** Characteristics of thank you letters in content analysis (N=100).

Characteristic			n (%)
**Author**			
	**Patient**		
		Male	14 (14)
		Female	38 (38)
		Unspecified	28 (28)
	**Relative**		
		Male	2 (2)
		Female	4 (4)
		Unspecified	13 (13)
	**Other**		
		Male	1^a^ (1)
**Type of institution, unspecified**			
	University hospital or medical center		12 (12)
	Regional or community hospitals		55 (55)
	Clinic or specialty center		33 (33)
**Location, unspecified**			
	United States		84 (84)
	United Kingdom		16 (16)

^a^This letter was from a military commander thanking a neurosurgeon for his work to advance treatment and asking for advice to help take care of wounded soldiers under his command.

### Coding Content

Patient evaluations and the measures capturing them can be narrow or broad. This determines how indicative they are of merit or flaw. As noted by Cleary and McNeil, “global measures of satisfaction are affected by many factors, and unless a study is conducted in extremely well controlled circumstances, it is difficult to interpret global measures” [9]. This implies that measurement of specific care domains is to be preferred over the measurement of broader ones. Therefore, not to lose information from letters in our study, we searched both for broad global-category evaluations (eg, access) and for narrow subcategory-specific evaluations (eg, within access: “responsiveness to phone calls”).

Each sentence or utterance within a sentence was marked as a thank you for one or more of the aspects of interactions between patients and their medical care providers. To do this, we used a coding rubric that was adapted from the Anderson taxonomy [4].

Our initial attempt to code the thank you letters further identified statements that did not clearly correspond to the operational definitions and examples originally proposed by Anderson and colleagues [4] for rating physician visits and required some adjustments. For example, a statement such as ‟your gentle staff were great” does not fall into the office staff or coordination as defined by Anderson [4], with a bureaucratic emphasis. In these cases, we analyzed the sentence according to content, not according to the object of praise. So, with the example above, we would code “your gentle staff were great” as praise for personality or demeanor.

To encompass all the sentences included in the thank you letters, we broadened the scope of several main categories and added several new subcategories to the Anderson [4] coding scheme. Within medical care, we added the subcategory treatment outcome, which gives the long-term perspective of treatment. To illustrate: (about a baby who had liver transplant), “Now Dean truly is...a good size and growing fast, a typical toddler.” We also added the subcategory “friendliness to the personality or demeanor category and the subcategory availability to the access category. Furthermore, as some utterances did not fall within any of Anderson’s, we added two categories to the classification. These were general praise (eg, “Both of my parents are lucky to be patients of Dr. Tendler”) and other for statements that lacked any form of praise (eg, “I recently had Mohs surgery with Dr. Makkar”).

Additionally, whereas Anderson [4] had a subcategory of general in the access, communication and personality or demeanor categories, we added general to all other categories. For example, a statement such as “Tammy (Sachowsky) was great on my first mammogram” was coded as general under medical care, as this does not clearly correspond to any of its specific subcategories. See Multimedia Appendix 1 for a final list of the categories and subcategories, along with operational definitions and examples.

Each sentence or utterance within a sentence was coded to indicate the presence (1) or absence (0) of content corresponding to each of the categories and subcategories represented in final coding scheme. Two coders independently coded the content of the first 63 letters that contained 415 sentences in total. Discrepancies were discussed and resolved through dialogue between the 2 coders; because interrater reliability was very high, as indicated in the Results section, the remaining 37 letters were then coded by one coder, and the coder’s ratings for the complete sample of 100 letters were retained for content analysis.

## Results

### Interrater Agreement

Several measures of interrater agreement were calculated to verify the coding scheme. For each category, the percent agreement in scores between the raters—that is, the total number of matches in ratings between the raters, indicating either the presence or absence of content in each utterance, divided by the total number of ratings for the content category—was very high, ranging between 90% and 100%. Interrater reliability (.84) was measured using the index of reliability [14]. Interrater reliability, as measured by Cohen kappa [15], was also substantial (.67), albeit lower, because of the extremely low prevalence of content for the categories of continuity, which was indicated for only four and seven utterances by the 2 raters, respectively, and administrative coordination, which was indicated for only one and two utterances by the 2 raters, respectively. Indeed, one limitation of kappa is that when the prevalence of a phenomenon in question is quite low, discrepancies between observed agreement and kappa can be very high [16,17]. By contrast, the index of reliability also provides a measure of interrater precision that takes expected levels of chance agreement into account without depending upon the marginal frequencies (ie, prevalence) of the content categories, and as such, it is arguably more appropriate for this analysis.

### Content Analysis

The content analysis sample consisted of 100 letters in total selected from 26 Web-based sources, corresponding to 26 medical facilities. On average, there were six sentences per letter, and 22 sentences were coded per source. Whereas the vast majority of sentences (76%, or 435 sentences) received a rating for only one content category, 22% (or 126 sentences) were coded in two to three content categories, and the remaining sentences received ratings across four to seven different content categories. Thus, sentences received 1.4 ratings, on average, across the content categories.

**Figure 1:**
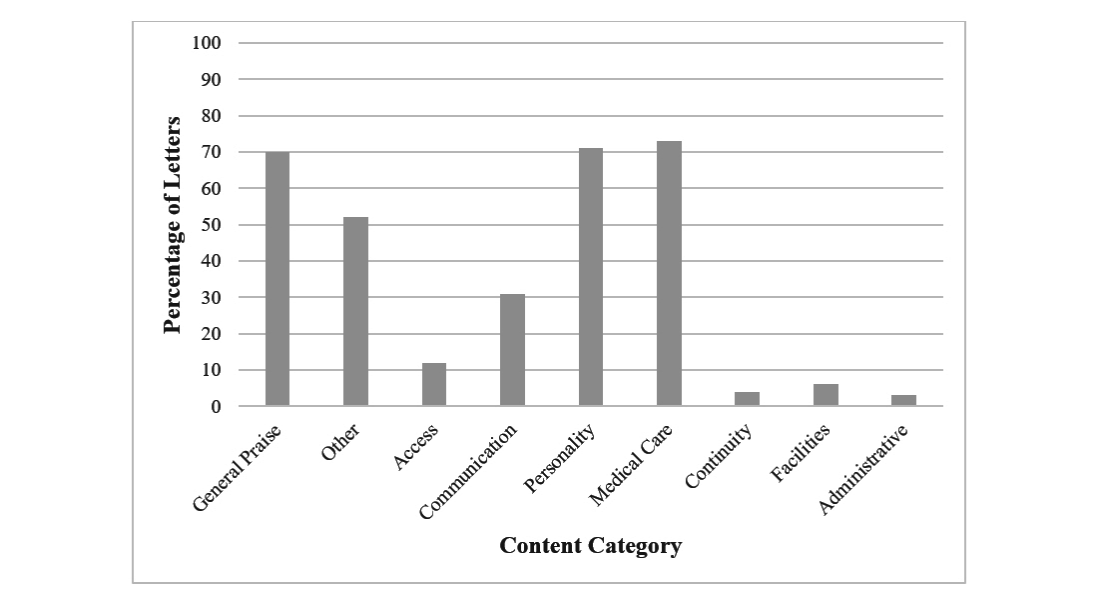
Percentage of thank you letters with content indicating various aspects of interactions with medical care providers.

Figure 1 shows the percentage of letters that express at least one utterance for each of the major content categories. Letters tended to express the most gratitude for the medical care provided, followed by gratitude for personality and demeanor and nonspecific general praise. Most patients expressed praise for these three categories, with 73%, 71%, and 70% expressing gratitude for medical care, personality, and nonspecific general praise, respectively. Other was the next most frequent category mentioned, with 52% of letters with many factual statements, for example, “I recently had surgery with Dr. Smith.” Patients tended to express less gratitude for the communication skills of their medical care providers, with only 31% of letters. Finally, patients seldom expressed gratitude for facilities (6 letters), access, (12 letters), continuity (4 letters), and administrative coordination (3 letters). Indeed, a chi-square test of goodness-of-fit confirmed that the number of letters varied with respect to the type of content expressed, χ^2^_8, N=900_=331.6, *P*<.01. This analysis was based on constructing a 9 (content category) x 2 (expression of gratitude: yes vs no) contingency table with all 100 letters represented in each column, totaling 900 observations.

Offering a more detailed view, Table 2 reports total number of utterances, the percentage of letters with content in that category, and the proportion of letters with general or specific utterances within each major content category. Letters differed in their tendency to express gratitude for general versus specific aspects of interactions with medical care providers. When praising the personality or demeanor of their medical care providers, patients referred mostly to specific qualities such as being supportive and understanding, 71.8% (51% of letters/71% of letters within category), humane and caring, 68% (48/71), or friendly, 28% (20/71). Fewer letters gave general praise to medical care providers’ personality or demeanor, 25% (18/71). By contrast, when referring to medical care, letters referred more to general qualities, 71% (52/73). The specific quality of medical care that was mentioned the most was treatment outcomes, 41% (30/73). This was followed by technical competence, 20.5% (15/73) and treatment approach 19% (14/73). In view of supportive being the most highly endorsed subcategory within personality and medical outcomes being the most highly rated medical care subcategory, it appears that people are most thankful for tangible outcomes—and processes—of their treatment. Chi-square tests of independence were performed to examine the relationship between these two types of content (personality and medical care) and the specificity of utterances in thank you letters. Although no significant differences were observed in the number of letters that expressed gratitude for the personality or demeanor of medical care providers as compared with the quality of their medical care (χ^2^_1,N__=200_=0.1, not significant), letters tended to express more specific praise for personality or demeanor but more general praise for medical care (χ^2^_1,N__=424_=63.9, *P*<.01). The chi-square analyses were based on constructing a 2 (content category: personality or demeanor vs quality of medical care) x 2 (expression of gratitude: yes vs no) contingency table with all 100 letters represented in both columns and rows, totaling 200 observations, and based on constructing a 2 (content category: personality or demeanor vs quality of medical care) x 2 (specificity of praise: general vs specific) contingency table representing the number of utterances expressed across letters for each content category and level of specificity, totaling 424 observations.

**Figure 2:**
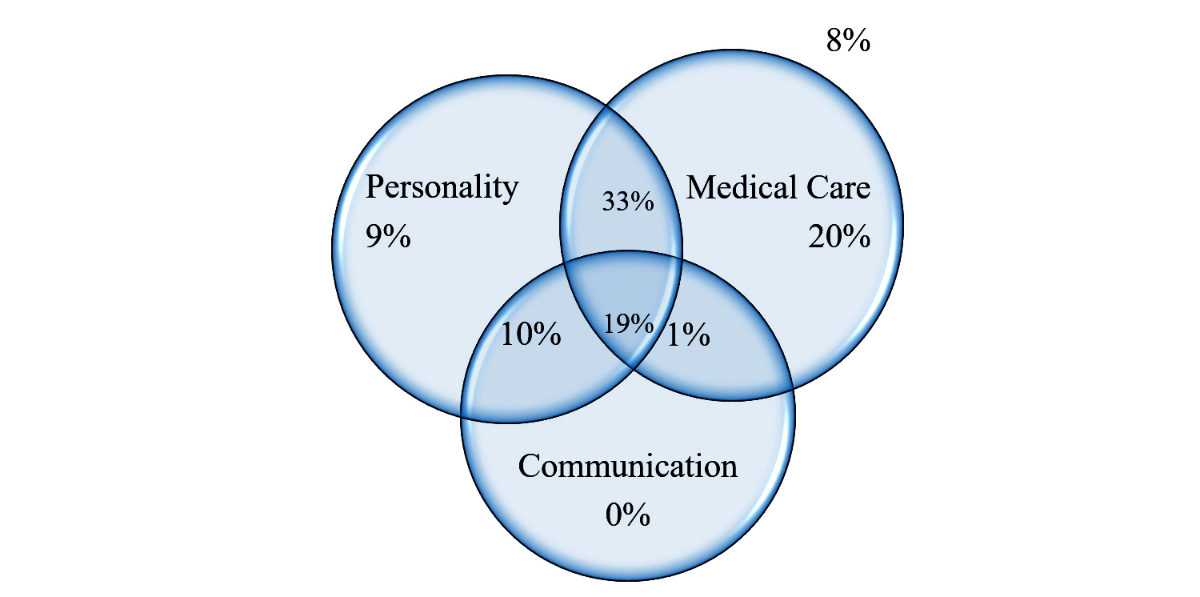
Proportion of letters that mentioned each topic. Percentages are out of 100% (N=100) letters and testimonials analyzed.

Figure 2 examines the juxtaposition of categories included per letter. People tended to simultaneously express praise for both personality or demeanor and medical care (33%) or for the personality, communication skills, and medical care aspects of their experience (19%). A large proportion of letter writers expressed thanks for the medical team’s medical care (73%), of which many expressed thanks for medical care and communication with them (20% of all letters). A similar proportion of letter writers (71%) expressed thanks for personality or demeanor, of which many expressed thanks for personality or demeanor and communication (29% of all letters). The surprisingly similar proportions of references to personality or demeanor and to medical care help validate the point that, to patients, these two are equally important. Interestingly, a relatively small percentage of letters (29%) only referred to a single major aspect, such as personality or medical care. A few (8%) of the letters did not refer to medical care, personality or demeanor, or communication.

**Table 2:** Total number of utterances, the number of letters with content in that category, and the proportion of letters with general or specific utterances within each major content category.

Content category			Total number of utterances (percentage of utterances within category)	Number of all letters (percentage of letters within category)
**Personality**				
	General		28 (12.1)	18 (25)
	**Specific**			
		Friendliness	29 (12.6)	20 (28)
		Humaneness and caring	67 (29.0)	48 (68)
		Supportive and understanding	89 (38.5)	51 (72)
		Trust	7 (3.0)	6 (9)
		Family or child	11 (4.8)	7 (10)
	Total		231 (100)	71 (100)
**Medical care**				
	General		91 (47.2)	52 (71)
	**Specific**			
		Patient advocacy	0 (0.0)	0 (0)
		Technical competence	22 (11.4)	15 (21)
		Time spent with patient	4 (2.1)	3 (4)
		Diagnostic skills	5 (2.6)	5 (7)
		Treatment approach	18 (9.3)	14 (19)
		Thoroughness	1 (0.5)	1 (1)
		Treatment options	1 (0.5)	1 (1)
		Treatment outcomes	51 (26.4)	30 (41)
		Providing medications	0 (0.0)	0 (0)
	Total		193 (100.0)	73 (100)
General praise			143 (100.0)	70 (100)
Other			130 (100.0)	52 (100)
**Communication**				
	General		3 (7)	3 (10)
	**Specific**			
		Listening skills	11 (24)	11 (36)
		Patient as partner	6 (13)	6 (19)
		Giving information	26 (57)	22 (71)
	Total		46 (100)	31 (100)
**Access**				
	General		0 (0)	0 (0)
	**Specific**			
		Waiting times	1 (8)	1 (8)
		Responsive to phone calls	2 (17)	2 (17)
		Availability	9 (75)	9 (75)
	Total		12 (100)	12 (100)
**Facilities**				
	General		3 (33)	2 (33)
	Specific		6 (67)	5 (83)
	Total		9 (100)	6 (100)
**Continuity**				
	General		0 (0)	0 (0)
	**Specific**			
		Follow-up care	4 (80)	4 (100)
		Test results	1 (20)	1 (25)
		Referrals	0 (0)	0 (0)
	Total		5 (100)	4 (100)
**Administrative**				
	General		2 (50)	2 (67)
	Specific		2 (50)	2 (67)
	Total		4 (100)	3 (100)

Patients expressed gratitude for personality and medical care together in 52% of the letters analyzed, and they expressed gratitude for personality, medical care, and communication in 19% of the letters analyzed. Only eight of the letters (ie, 8%) lacked reference to medical care, personality or demeanor, or communication. Six letters mentioned nonspecific general praise or statements that did not indicate any form of praise (eg, other), and 2 letters expressed praise for the medical facilities.

## Discussion

### Principal Findings

The primary objective of this study was to address the question—what aspects of care do patients thank doctors and medical facilities for? Our main finding is that, first and foremost, patients and family members are thankful for their providers’ medical care. Second, and very closely related, they are thankful for personality and demeanor.

Thus far, physician ratings, patient forums, and other Web-based resources provide insights to people’s opinions of, and points of dissatisfaction with, their medical care. For example, a recent comparison of women’s posts on pregnancy in general versus on vaginal birth after cesarean (VBAC) unveiled women’s greater need for information and emotional support in the case of VBAC [18]. Our findings correspond with those of Lagu et al, who analyzed 33 physician-rating websites. Patients were asked to provide feedback on what the authors labeled as clinical aspects, which included physician’s knowledge (49%), bedside manner (39%), and communication skills (30%). Nonclinical aspects involved punctuality (49%) and staff friendliness (36%) [19].

The analysis of thank you letters to physicians and medical centers offers additional resource relating to how patients view health care, which has thus far not been investigated. When it comes to the topic of medical care, letters are more likely to include general words of praise than any other category. Whereas some general praise is also awarded for physician personality, an overwhelming majority of the letters in this category mentioned the physician’s supportiveness. This is one element of care that is both tangible to patients and appreciated by them.

Patients’ abilities to point to specifics in their doctors’ personalities or demeanors and then secondarily point to good medical care in general is reflected in the paper’s title, which comes from a patient’s letter, the full citation being, “She is just a phenomenal person and doctor,” in this order. In more than half of the cases, these two aspects are mentioned together in the same letter, suggesting that from the perspective of the patient and family member, both are deemed necessary to evaluate care positively. The centrality of the emotional aspects of care dovetails with previous findings [13].

This somewhat mirrors the proportion of these aspects in patients’ free form writing about physicians, indicating mainly that medical care per se, alongside bedside manners and communication, are what patients consider the bedrock of good treatment.

We looked at patients’ and family members’ responses to mostly successful outcomes, finding that patients deem providers’ personality and behavior highly important, considering them more than the technical aspect of their professional actions. Similar principles, albeit in a mirror image, apply when observing malpractice claims. A survey of patients and relatives who took legal action against doctors found that the decision of patients and relatives to take legal action against their providers was determined not only by the original injury but also by what they perceived as insensitive handling and poor communication after the original incident [20]. Indeed, several studies suggest that doctors with better communication skills or even a tone of voice that is considered kinder, are less likely to be involved in malpractice claims [21,22]. This suggests that negative sentiment toward the medical staff is a major component in patients’ overall dissatisfaction, rather than an adverse outcome per se. All these highlight the role of the interpersonal connection in medicine.

Most medical schools have implemented courses for training clinical communication skills. Indeed, data support improved communication behaviors among providers following such educational interventions [23]. However, it seems that postgraduate communication training for doctors is lacking [24].

### Limitations

Several limitations exist in our investigation. For the purposes of our investigation, it is important to acknowledge the influence of selection bias in our content sample—not because of any of our practices but rather in accordance with the centers’ interests. The letters and testimonials were posted by the medical centers themselves, and so they likely only posted letters and excerpts in which the content was consistent with the way the centers wished to be portrayed to the general public. As a result, the medical centers may have refrained from posting any letters that included negative statements about their staff or facilities or may have censored negative statements from posted material. On the other hand, as the focus of this work is the thank you letter, we were less concerned with letters of complaint. We also could not verify the authenticity of the letters themselves and relied on the assumption that the medical centers exercised professionalism by only posting authentic letters or testimonials. In this respect, the letters might be representative not only of how patients evaluate their care but also of how doctors and medical centers wish to be perceived by prospective patients and perhaps of what they feel such patients can comprehend and appreciate. This is a form of selection bias—of what is displayed on the Web.

There may also be a degree of selection bias in the types of letters that patients themselves send to medical centers and agree to have publicized on the Internet. When writing personal thank you letters to physicians, patients may prefer to write and deliver a letter by hand to make the gesture of appreciation more direct and personal. Such letters may also be accompanied by physical gifts (eg, box of chocolates and alcohol). By contrast, letters that patients send to medical centers may be more intended to give wider recognition to their physicians and other medical staff involved in their treatment.

Another limitation is the way the sample was created—albeit this was done based on the most prominent search results, wishing to cover as many specialties as possible and to only analyze several letters from each center so that no particular center’s results are overrepresented. A more extensive inquiry, following this exploratory investigation, would perhaps include all the letters posted on the Web by all the centers from a specific specialty, in a specific geographical region.

The data analysis in this pilot work on thank you letters was strictly descriptive. We chose not to use significance testing, as the coding scheme involved seven categories, some of which resulted in few observations. Thus, a pairwise comparison between medical and personality or demeanor praise would prove problematic because of the Bonferroni corrections required. An analysis of variance, on the other hand, would merely show that differences exist. Future investigations of a larger scale can proceed with significance testing and planned comparisons.

### Conclusions

Despite these limitations in selection and analysis, the texts provide a useful dataset for characterizing which aspects of medical treatment are considered important to patients and their families. It is possible that the letters also provide a glimpse into what matters to medical care providers, which is important for them to convey to future patients who might be reading these letters on the Web. This would be in line with the concern that social media is viewed as a marketing tool or as a means of engaging in a meaningful conversation with patients [3]. Future projects should in more depth analyze larger samples of letters, also taking into account nonpublished notes. As new ways of care evolve in a digital age, this information may help to better address communication needs of patients. In addition, to prove the validity of our method, a comparison with existing methods and surveys (MISS and CSQ) should be undertaken.

Limitations notwithstanding, these letters demonstrate the strong emphasis that hospitals, clinics, and medical educators should place on ensuring personable care, which is so crucial for patients’ experiences. If letters are not just posted for display but also read and learned, they can be a powerful beacon guiding medical care providers toward patient satisfaction.

## Appendix

Multimedia Appendix 1 Coding scheme adapted from Anderson et al (2007).

## Abbreviations

CSQ: Consultation Satisfaction Questionnaire
MISS: Medical Interview Satisfaction Scale
VBAC: vaginal birth after cesarean
