# Supplementary material for: “A Phenomenal Person and Doctor”: Thank You Letters to Medical Care Providers
Source: Interact J Med Res. 2017 Nov 2;6(2):e22. doi: 10.2196/ijmr.7107 (PMC5695918; doi:10.2196/ijmr.7107)
Supplement: Multimedia Appendix 1 [file ijmr_v6i2e22_app1.pdf]

Appendix: Coding Scheme Adapted from Anderson, Barbara, and Feldman (2007)

Access
Definition from Anderson et al. (2007): "This category emphasizes the ability to make an appointment or see or contact a physician or other provider in a timely manner. Includes both office visits and telephone contact.

Wait time
- "He keeps appointments on time or very close."
- "Always timely."

Phone
- "She will personally call you at home with test results."
- "You responded to ***all our phone calls***, questions and concerns so promptly and patiently."

Availability
- "She makes herself available and accessible beyond her working hours."
- "I was brought in by ambulance & was able to see the same ER dr. that I saw at the Silverdale Harrison ER the prior night."

Communication
From Anderson et al. (2007): "The category measures the style and manner in which the provider verbally communicates to the patient. It involves four distinct and essential qualities, with the goal of including the patient in the healthcare process."

Listening
- "He truly listens and does not shrug off what you are feeling."
- "She always seems to have time to listen and give me her full attention."

Patient as Partner/Shared Decision-Making (SDM)
- "He allows the patients to be an active participant in healthcare decision-making."
- "Thank you so much for making this emotional decision as easy as possible for me."

Giving Information
- "She drew pictures, explained everything in great detail."
- "My surgeon was also very patient with questions from my family…"
-

Personality/Demeanor
From Anderson et al. (2007): "This category reflects the provider's general behavior and disposition toward the patient, involving [five] distinct traits that convey empathy and caring. This can include both verbal and nonverbal behaviors, and extends beyond the patient to include the family."

Friendliness
- "We pulled up to the main entrance and were greeted by two young men (Valets) who welcomed us with smiles and offered to valet park our cars while the surgery was done."
- "His manner is friendly, dignified, well-mannered, and pleasant."

Humaneness/Caring

- “She is a wonderful person with a kind and caring heart.”
- “He is very compassionate and makes me more at ease during my visits.”

Supportive/Understanding

- “I felt that I was important to every staff member in the pain center and that I would be treated exceptionally well.”
- “As I was recovering on the third floor, the nurses were very caring, reassuring me I would be fine.”

Trust

- “I knew at that point I had no worries.”
- “It’s important to make patients feel comfortable and trusting in your ability to take care of them.”

Family/Children

- “Very attentive to both parent and child.”
- “He lets you know that he cares about the whole family by working hard to know everyone personally.”

Medical Care

From Anderson et al. (2007): “This large category includes [nine] aspects of medical care that patients recognize as important and value. These range from the skill level of the physician and thoroughness of care to the overall treatment approach of diagnosis, treating, and following up on results.” This domain also refers to individuals' reactions to their or their family members’ short- and/or long-term outcomes of medical treatment.

Advocate

- “He will go to bat with the insurance company if they deny medication.”
- “Willing to fight for her patients’ needs.”

Technical Competence

- “While there, the nurses and Dr. Enloe worked so fast.”

Time spent with patient

- “I tell them how they take their time with you…”
- “The technician took the time to sit with me while I waited for an ultrasound.”

Diagnoses

- “We will be forever grateful to you for finding my carcinoid tumor…”
- “I am also grateful that he suggested removing the other organs because it seems the Histology staff found traces of a few malignant cancer cells in the uterus which we were totally unaware of, which had also been removed in the operation, follow-up checks have been arranged, so I suspect his decision had basically saved my life, in more ways that we originally thought.

Treatment approach

- “Besides, he started me with two visits per month where he would do first acupuncture and after that treatment he would do trigger point injections in the muscle area to get those knots of tension out.”
- “We cannot say enough about the way he was treated and the care he was given by everyone.”

Thoroughness

- "Very thorough."
- "She is extremely thorough and conscientious."

Treatment options

- "He is willing to entertain options of alternative treatments and has helped us research nutritional supplements."

Treatment outcome

- "I always meant to send a thank you letter, but now that it is his 1-year anniversary and we can see how much he has changed, what you have done for us is even more meaningful."
- "We will be forever grateful to you … for helping us to conceive."

Medications

- "Only prescribes medicine when necessary."
- "If you don't have a good prescription plan, he has plenty of samples."
-

Continuity

From Anderson et al. (2007): "This separate domain looks at how information is passed from one visit to the next and from one provider or practice to the next so that care is planned and integrated across time and place."

Follow-up

- "He checks up on a regular basis if you've not been doing well."

Test results

- "I tell them how …. quickly they get you through your test and how they do it without any inconvenience to you or without jeopardizing the quality of the test or the outcome.

Referrals

- "Very good about follow-up with other doctors involved."

Facilities

From Anderson et al. (2007): "This category is narrowly defined as the look and feel of the physical location or facility of the office or clinic."

- "I like the fact that the glass was removed from the reception desks."
- "Our first impression was that we were pulling up to a premier hotel, not a hospital."

Administrative Coordination

From Anderson et al. (2007): "This category addresses support staff that assist the patient's access to medical care and may serve a critical role as a liaison between patient and doctor."

- "Very well-run office."
- "Her office staff are excellent and return and answer calls."
